# Herbivory on Temperate Rainforest Seedlings in Sun and Shade: Resistance, Tolerance and Habitat Distribution

**DOI:** 10.1371/journal.pone.0011460

**Published:** 2010-07-07

**Authors:** Cristian Salgado-Luarte, Ernesto Gianoli

**Affiliations:** 1 Departamento de Botánica, Universidad de Concepción, Concepción, Chile; 2 Departamento de Biología, Universidad de La Serena, La Serena, Chile; 3 Center for Advanced Studies in Ecology and Biodiversity, P. Universidad Católica de Chile, Santiago, Chile; Centre National de la Recherche Scientifique, France

## Abstract

Differential herbivory and/or differential plant resistance or tolerance in sun and shade environments may influence plant distribution along the light gradient. *Embothrium coccineum* is one of the few light-demanding tree species in the temperate rainforest of southern South America, and seedlings are frequently attacked by insects and snails. Herbivory may contribute to the exclusion of *E. coccineum* from the shade if 1) herbivory pressure is greater in the shade, which in turn can result from shade plants being less resistant or from habitat preferences of herbivores, and/or 2) consequences of damage are more detrimental in the shade, i.e., shade plants are less tolerant. We tested this in a field study with naturally established seedlings in treefall gaps (sun) and forest understory (shade) in a temperate rainforest of southern Chile. Seedlings growing in the sun sustained nearly 40% more herbivore damage and displayed half of the specific leaf area than those growing in the shade. A palatability test showed that a generalist snail consumed ten times more leaf area when fed on shade leaves compared to sun leaves, i.e., plant resistance was greater in sun-grown seedlings. Herbivore abundance (total biomass) was two-fold greater in treefall gaps compared to the forest understory. Undamaged seedlings survived better and showed a slightly higher growth rate in the sun. Whereas simulated herbivory in the shade decreased seedling survival and growth by 34% and 19%, respectively, damaged and undamaged seedlings showed similar survival and growth in the sun. Leaf tissue lost to herbivores in the shade appears to be too expensive to replace under the limiting light conditions of forest understory. Following evaluations of herbivore abundance and plant resistance and tolerance in contrasting light environments, we have shown how herbivory on a light-demanding tree species may contribute to its exclusion from shade sites. Thus, in the shaded forest understory, where the seedlings of some tree species are close to their physiological tolerance limit, herbivory could play an important role in plant establishment.

## Introduction

Herbivory is an important factor in plant ecology and evolution in forest communities [1]. Damage by herbivores may decrease plant performance and fitness [2], [3] and restrict plant distribution along the light gradient [4]–[7]. It has been shown for a number of plant species that plants sustain greater insect herbivory when growing in shaded habitats [8]–[14]. In contrast, there are several reports of increased herbivory in open sites [4], [15]–[20] or similar rates of herbivory in sun and shade [21]. Differential herbivory in contrasting light environments may reflect light-induced differences in plant defensive traits, such as leaf toughness and carbon-based secondary chemicals, or could result from differences in herbivore abundance between habitats [4], [22]–[27]. Therefore, in order to explain field patterns of herbivory across light environments it is necessary to include both herbivore palatability bioassays [28]–[31] and field estimates of herbivore abundance [19], [32], [33].

Studies addressing herbivory in sun vs. shade have often focused on plant resistance to herbivores. From a phytocentric perspective, however, it is essential to determine the actual consequences of herbivory for the plant in terms of performance, fitness, abundance or distribution [34]. It is assumed that a given amount of damage may cause greater fitness losses in shade than in sun [35]–[37]. This differential tolerance of herbivory is explained by the fact that in the shaded understory, where carbon gain is low, compensation of photosynthetic tissue lost to herbivores is more expensive in terms of resources and time. Light availability limits plant performance and fitness in forest ecosystems [38]–[40] and herbivory may affect light exploitation by reducing leaf area or by constraining functional phenotypic responses of plants to shading (Salgado-Luarte & Gianoli, unpublished). While most studies have found lowered tolerance of herbivory in the shade [41]–[46], there are also cases of similar levels of tolerance with varying levels of light availability [44], [47]. It is rather surprising that the large majority of plant-oriented studies of herbivory in contrasting light environments has evaluated defensive traits in plants but rarely has included evaluations of plant tolerance. To better understand the effect of herbivores on plants along the light gradient and predict future scenarios of plant distribution, it is important to estimate plant tolerance of herbivory as well as plant resistance against herbivores.

Ecological theory relating resource availability and plant allocation to growth and defense, was originally formulated at the interspecific scale [36], [48], but it has also been applied at the within-species scale [49]–[51]. It posits that lowered tolerance in the shade should be counteracted by a greater resource allocation to chemical defenses that would confer increased resistance against herbivores, thus avoiding damage. Therefore, the greater fitness impact of damage in the shade constitutes the selective scenario for the evolution of increased allocation into defense and ensuing plant resistance. In general, if a plant species has decreased tolerance but increased resistance in the shade, or vice versa, it may be able to survive in shaded habitats. This assumes that plant resistance and tolerance are defensive strategies of comparable efficiency in the struggle against herbivores [52]. If a plant species exhibits both decreased tolerance of damage and lowered resistance to herbivores in the shade as compared to open sites, conditions are given for exclusion from shaded habitats by herbivores. This cannot be inferred solely from field patterns of herbivory in sun and shade. The first demonstration of herbivory as a key factor explaining the distribution of a plant species along the light gradient was published by Louda & Rodman in 1996 [4], and little empirical evidence on this issue has accumulated since then [5], [7], [53].

In the southern temperate evergreen rainforest most tree species show intermediate levels of shade-tolerance [54], [55]. One of the few species considered light-demanding is *Embothrium coccineum* (Proteaceae) [40], [54], a small tree endemic to South American temperate forests that is commonly found in open sites [56]. Comparatively high mortality of *E. coccineum* in shaded sites was hypothesized to be related to negative carbon balance, but there were also observations of defoliation by invertebrate herbivores [54]. We have observed several individuals of *E. coccineum* of vigorous appearance in the shade and then the question arises whether herbivory plays a role in the habitat distribution of this endemic species. Herbivory may contribute to exclude *E. coccineum* from the shade if 1) herbivory pressure is greater in the shade, which in turn can be related to plant resistance, i.e., shade plants are less resistant, or to habitat preferences of herbivores, and/or 2) consequences of damage are more detrimental in the shade, i.e., shade plants are less tolerant.

The first objective of this study was to evaluate and explain the pattern of herbivory on *E. coccineum* seedlings in treefall gaps (sun) and forest understory (shade). To determine whether differential herbivory in sun and shade reflected light-induced differences in plant resistance or differences in herbivore pressure, we tested the palatability of sun and shade leaves with a generalist herbivore and estimated herbivore abundance in both light environments. The second objective was to compare plant tolerance of herbivory in sun and shade. We simulated herbivore damage on seedlings established in sun and shade and afterwards evaluated seedling survival and growth. Experiments allowed us to test predictions regarding the relationship between plant resistance and tolerance in the shade and the observed habitat distribution of *E. coccineum*.

## Methods

### Field Patterns of Foliar Herbivory in Sun and Shade

Sampling was carried out in the mature temperate rainforest at Puyehue National Park (40°39′S, 72°11′W; 350–400 m a.s.l.), in the western foothills of the Andes in southern Chile. The study site (Anticura) has an annual precipitation of 2800 mm and a mean temperature of 9.8°C [57]. The old-growth lowland forest is composed of broad-leaved evergreen trees [54], [58] and woody vines [59]. With regard to light availability, the study area is strongly skewed towards low light, with 43% of microsites occurring at 5% canopy openness and microsites with >25% canopy openness being rare [55]. Two contrasting light environments were chosen for estimations of herbivory: treefall canopy gaps (sun) and mature forest understory (shade). To characterize the light environment, we selected three sites at least 2 km apart in both sun and shade and conducted several measurements of photosynthetic active radiation (PAR) at noon with a LI-250 Light Meter (LI-COR). Light availability was similar among sites within each light environment (P>0.15, Kruskal-Wallis ANOVA, data not shown) and markedly differed between sun and shade (P<0.001, Mann-Whitney U test, data not shown). Canopy gaps and forest understories received about 67% and 5% of full sunlight, respectively.

We sampled 68 and 64 seedlings of *E. coccineum* in sun and shade, respectively. Seedling height ranged from 20 to 40 cm and they were at least 5 m apart. For each seedling we selected at random five leaves to estimate the magnitude of herbivory. Each of the five leaves was assigned to one of the following categories of damage, based on visual inspection of leaf area removed: **0**, no damage; **1**, less than 25% damage; **2**, from 25% to 50% damage; **3**, from 50% to 75% damage; and **4**, damage above 75%. The score of all leaves was used to calculate an individual index of herbivory, IH = **Σ** ***nC***_0–4_ ***N*** ^−1^; where ***C*** is the category of damage, ***n*** is the number of leaves in the ***C***th category, and ***N*** is the number of leaves sampled (five, in this case) [60]. Similar indices have been used in earlier studies [19], [61]. We collected one intact leaf from a separate set of 20 seedlings in both sun and shade environments to determine the specific leaf area (SLA, cm^2^ g^−1^). We compared IH between sun and shade using a t-test. Furthermore, to make a comparison including a gross estimation of herbivory standardized by herbivore abundance, we used a sun vs. shade 2×2 table of contingency, where IH was the numerator and total herbivore biomass (see below) was the denominator. Differences in SLA between sun and shade were evaluated with a t-test.

### Leaf Palatability (Plant Resistance)

To determine whether plant resistance differs between light environments, a no-choice test was conducted in a hut located in the study site. Because leaf damage in *E. coccineum* was caused both by insects and snails (Salgado-Luarte, personal observations), the generalist snail *Helix aspersa* was used as model herbivore. A total of 32 individuals of *H. aspersa* of similar size (5.8±1.0 g) were placed singly into 20 cm ×10 cm plastic boxes, and were starved during 48 h. We then placed a freshly collected leaf of *E. coccineum* in each box: 16 sun leaves (from seedlings in canopy gaps) and 16 shade leaves (from seedlings in forest understory) of similar size. All leaves used in the assay were fully expanded leaves at the time of collection and were taken from the mid-part of seedlings. The boxes were placed close to a window inside the hut and hence the test was conducted under light conditions intermediate to those found in the field. We took digital pictures of all 32 leaves twice, just before snails were put into boxes and 48 h later, when the test ended. We quantified leaf area consumed by snails (cm^2^) by comparing pictures taken at the beginning and at the end of the assay, using image analysis software (Sigma Scan Pro). To compare the palatability of plants grown in sun and shade (our measure of plant resistance), a Kruskal-Wallis test was used.

### Herbivore Abundance

To estimate herbivore abundance in sun and shade, we collected epigeous fauna at three times during late spring-early summer (November 2008, December 2008, and January 2009) in five canopy gaps and five forest understories within Puyehue National Park. At each of the ten sites, four pitfall traps (300 cc), containing a solution of 90% ethanol and detergent, were buried in the ground for walking or crawling herbivores to fall down. We collected the samples and renewed the trap solutions every 30 d. In addition, we used the beat sheet method to collect herbivores feeding or resting on plants. Five shrubs or saplings were beaten at 1–1.5 m height ten times with a 1 m wooden stick in each site, and arthropods and gastropods were collected over a fabric sheet (80 cm ×80 cm) placed behind the sampled plants. Summarizing, we sampled 75 plants per habitat (5 sites ×5 plants per site ×3 independent samplings during the season) and also placed and evaluated 60 pitfall traps per habitat (5 sites ×4 traps ×3 independent samplings during the season). Subsequently, all samples were oven-dried at 72°C and weighed. Although we did not determine the feeding habit of all collected specimens, it was evident that most of them were herbivores. Thus, the most abundant insects in the samples were leaf beetles, weevils and caterpillars, and snails were also frequently found (data not shown). Therefore, it was deemed reasonable to label “herbivore biomass” the total dry sample obtained. We compared herbivore abundance between sun and shade using a Mann-Whitney U test. There are no vertebrate herbivores in this temperate rainforest.

### Field Trial (Plant Tolerance)

To compare plant tolerance in sun and shade, we conducted a simulated herbivory experiment with *E. coccineum* seedlings naturally established in treefall canopy gaps (sun; average light intensity at noon: 952 µmol m^−2^ s^−1^ PAR) and mature forest understory (shade; average light intensity at noon: 64 µmol m^−2^ s^−1^ PAR) in Puyehue National Park. In September 2007, we selected 70 seedlings growing in sun and 70 seedlings growing in shade, arranged into seven groups of 10 seedlings each. Groups were defined by spatial proximity. Mean ± SE seedling height and leaf number were 24.8±0.4 cm and 8.2±3.1, respectively, and did not differ between sun and shade (data not shown). This size roughly corresponds to two-year old seedlings [56]. Half of the plants in each group (total n = 35 seedlings per light environment) were randomly assigned to receive simulated herbivory (50% damage), which consisted in cutting with scissors 50% of leaf area of all the leaves. This clipping treatment was repeated in March 2008 to maintain the level of leaf damage at 50%, which corresponds to the upper level of damage observed in *E. coccineum* seedlings in this forest as shown by the raw data used for the calculation of IH. Natural herbivores were excluded from both undamaged and experimentally damaged seedlings by treating them with systemic insecticide (Dimethoate plus, Fastac) and molluscicide (Metarex) monthly. In July 2008, 10 months after the onset of the experiment, we recorded seedling survival and estimated seedling relative growth rate as RGR =  (ln H_2_ - ln H_1_) t^−1^; where H_1_ and H_2_ are seedling height (cm) at the start and the end of the experiment, respectively, and t (days) is the time extent of the experiment. A two-way ANOVA, with Damage and Light as fixed factors, was used to compare seedling survival (arc-sin transformed) among seedling groups (seven replicates per light environment/damage treatment). A similar two-way ANOVA was applied for RGR (main factors: Light and Damage), but in this case we pooled data from all seedlings instead of considering averages for seedling groups, as in the case of survival. A Tukey HSD test was used for *a posteriori* comparisons.

## Results

**Figure 1:**
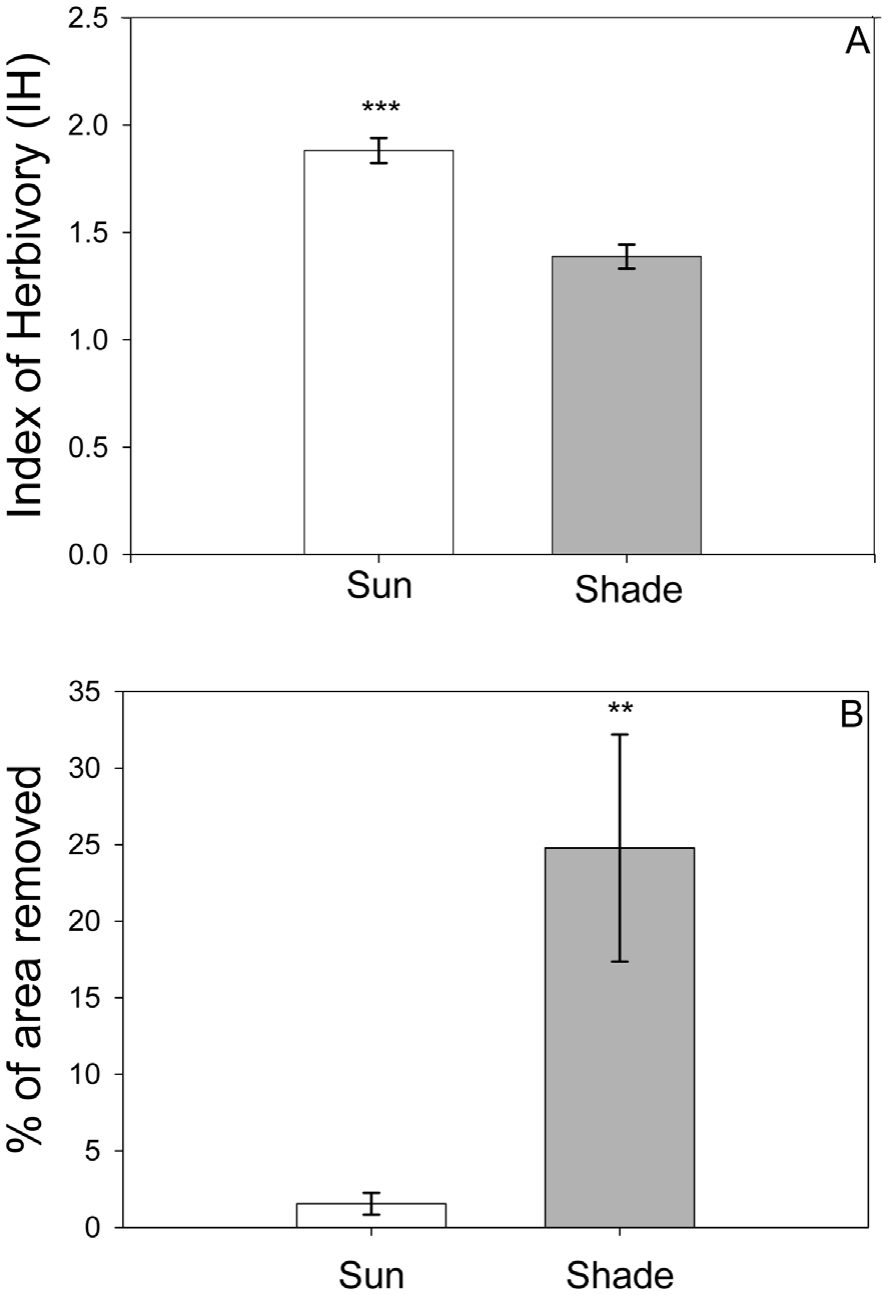
Herbivory pressure and plant resistance in *Embothrium coccineum* seedlings from contrasting light environments. A) Index of herbivory, IH (± SE) on seedlings in sun (white bar) and shade environments (gray bar) in a southern temperate rainforest. Means were significantly different (P<0.001; t-test). B) Leaf area consumed (% ± SE) by the generalist snail *Helix aspersa* in a 48 h no-choice palatability test with leaves from seedlings grown in sun (white bar) and shade (black bar) sites. Means were significantly different (P<0.005; Mann-Whitney U test).

Seedlings of *E. coccineum* growing in the sun sustained nearly 40% more herbivory damage than those growing in the shade (t_130_ = 6.11, P<0.001, t-test; Fig. 1A). In contrast, when plant damage was standardized by herbivore abundance, it was found that herbivores were more voracious in the shade than in the sun (χ^2^ = 5.38, P<0.025, 2×2 table of contingency). Shade seedlings displayed a greater SLA (cm^2^ g^−1^) than those growing in open sites (Sun: 154.1±2.7, Shade: 300.5±3.3, Mean ± S.E.; t_100_ = 34.18, P<0.001).

The palatability test showed that *H. aspersa* snails consumed around 10 times more leaf area when fed on shade leaves compared to sun leaves (U = 51.50, P<0.005, Mann-Whitney U test) (Fig. 1B). This indicates that plant resistance was greater in sun-grown individuals of *E. coccineum*.

Two-thirds of herbivore biomass harvested was from insects and one-third from small gastropods (data not shown). Herbivore biomass, our estimate of herbivore abundance, was almost twice greater in the sun (3.6±0.16 g) than in the shade (1.9±0.21 g) (U = 51.50, P<0.001; Mann-Whitney U test).

**Figure 2:**
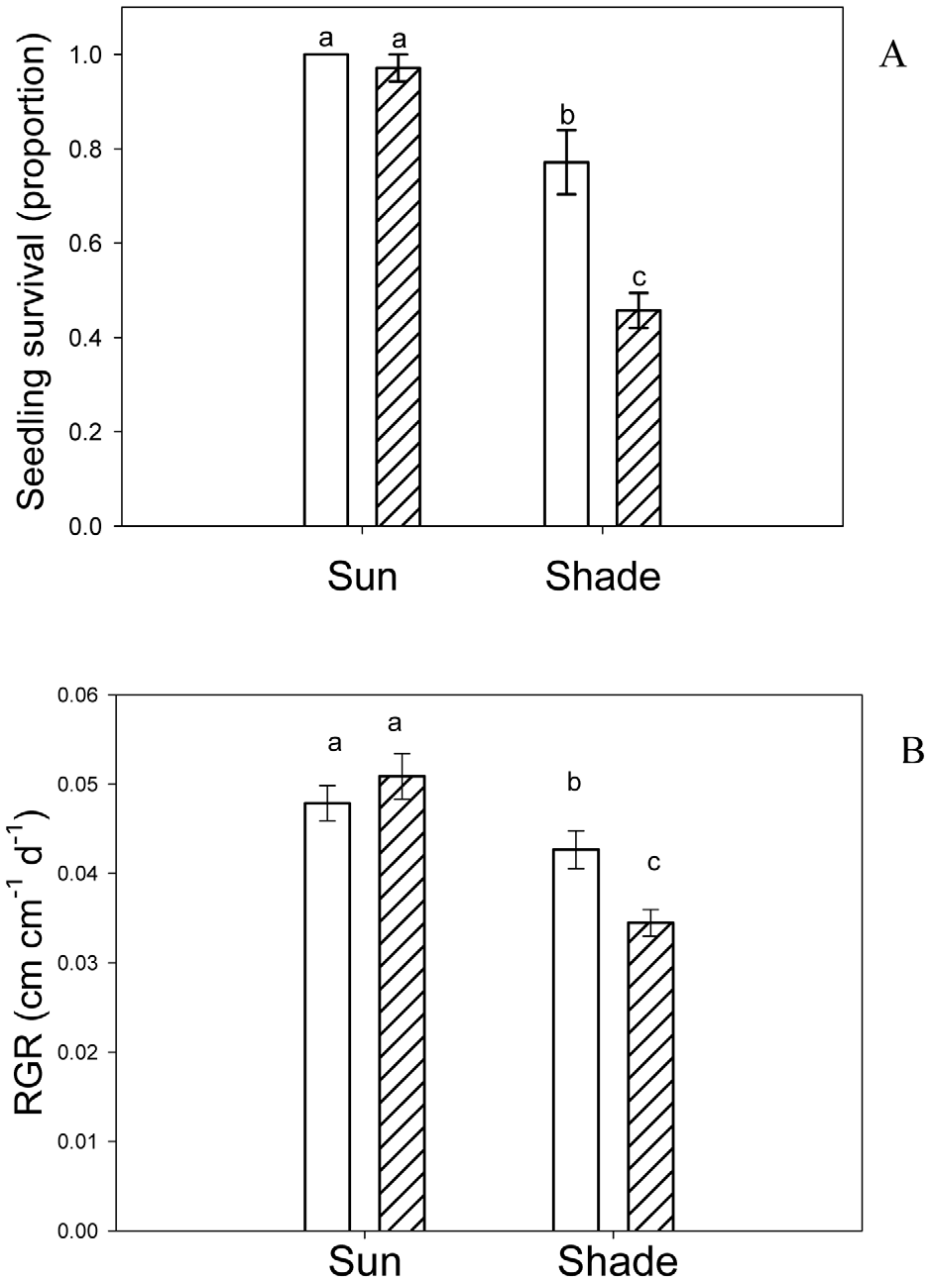
Effects of simulated herbivory on *Embothrium coccineum* seedlings in sun and shade sites. Open bars: undamaged seedlings; hatched bars: seedlings subjected to 50% leaf damage. Bars show results 10 months after inflicting damage. **A**) Seedling survival (proportions, ± SE). **B**) Seedling relative growth rate, RGR (cm cm^−1^ day^−1^, ± SE). Bars sharing a letter are not significantly different (Tukey HSD test).

Overall, seedling survival was greater in the sun and in undamaged plants, and seedling growth (RGR) was greater in the sun (Table 1). More related to our specific research question, there were significant Light × Damage interactions for both plant fitness components (Table 1). Whereas simulated herbivory in the shade decreased seedling survival and growth by 34% and 19%, respectively, damaged and undamaged seedlings showed similar survival and growth in the sun (Fig. 2). Undamaged seedlings survived better and showed a slightly higher growth rate in the sun than in the shade (Fig. 2).

**Table 1:** Analysis of variance of the effects of leaf damage and light environment on survival and growth of *Embothrium coccineum* seedlings.

	Survival		Relative Growth Rate	
Factor	F_1,24_	P-value	F_1,107_	P-value
Damage (D)	11.42	0.002	1.302	0.256
Light (L)	73.32	<0.001	22.62	<0.001
D × L	5.876	0.023	6.087	0.015

## Discussion

Seedlings of *E. coccineum* sustained greater herbivory when growing in treefall canopy gaps compared to the forest understory in this southern temperate rainforest. This pattern agrees with several studies reporting higher herbivory rates in open sites [4], [15]–[20]. Increased herbivory in the sun may result from light-induced differences in plant defensive traits, that in turn decrease plant resistance, or from differences in herbivore abundance between light environments [23]. Plant traits, even putatively defensive traits, do not always explain patterns of herbivory in contrasting light environments. For instance, Chacón & Armesto [19] found that seedlings of *Gevuina avellana* and *Drymis winteri* grown in treefall gaps in a temperate rainforest had higher levels of leaf phenols and tannins than those grown under closed canopy, but seedlings grown in gaps suffered greater leaf damage than those in forest interior. Likewise, Aide & Zimmerman [62] found that *Connarus turczaninowii* plants in open sites had lower concentrations of water and nitrogen, and were tougher, but no differences in herbivory rates were found in plants distributed along the light gradient in a tropical rainforest.

On one hand, results of the palatability bioassay indicate that plant resistance was greater in sun-grown plants, probably due to their thicker leaves (lower SLA). Plants with lower SLA often have tougher tissues that render them less palatable for herbivores [27], [63]–[65]. We cannot disregard, however, the involvement of other unmeasured defensive traits in the observed increased plant resistance (reduced palatability) of leaves from sun plants, as has been shown for chemical defenses in earlier studies [66]. Consequently, field patterns of increased herbivory on *E. coccineum* in open sites could not be explained by differential plant resistance. On the other hand, field estimations showed that herbivores were more abundant in open sites. These results are consistent with field herbivory patterns and thus suggest that the greater damage recorded on *E. coccineum* in the sun may be due to habitat preference by the main herbivores found in the forest (insects and snails). Differential abundance of herbivores in sun vs. shade may be explained by the interplay between abiotic factors (e.g., temperature, humidity) and biotic factors (e.g., food quality, natural enemies) that ultimately determine herbivore performance in each habitat [13], [16], [33], [67], [68]. Interestingly, working in the same forest ecosystem, the southern temperate evergreen rainforest, Chacón & Armesto [19] also reported a greater abundance of herbivores in canopy gaps than in forest understories, estimated as herbivore biomass.

Tolerance of leaf damage was lower in the low light environment. This result is consistent with specific predictions of the LRM [37] in the case that the resource that limits plant fitness and the resource whose use is primarily affected by herbivory are the same, i.e., light. A number of studies have reported lower tolerance of herbivory in the shade [41]–[46]. Summarizing, shade plants of *E. coccineum* exhibited both reduced resistance and tolerance compared to sun plants. Moreover, even though herbivores were more abundant in the sun, those causing damage in the shade were seemingly more active or voracious. Therefore, it could be suggested that herbivory pressure plays a role in the observed habitat distribution of *E. coccineum* in the southern temperate rainforest, where it is considered a light-demanding species [40], [54].

*E. coccineum* is a short-lived early successional species with a leaf life-span of approximately one year [69]. Because the 10-month survival of undamaged plants in sun was higher than that of undamaged plants in shade (100% vs. 80%), it is clear that –besides the biotic filter exerted by herbivores– there are intrinsic physiological constraints for the establishment of *E. coccineum* in shaded habitats. Lusk [54] remarked that the significant mortality of *E. coccineum* seedlings in the shade was not only due to a negative carbon balance, but also to defoliation by invertebrate herbivores. We undertook an experimental approach to this issue and found evidence of synergistic effects of herbivory and shade on *E. coccineum* fitness. Thus, herbivore damage decreased seedling survival and growth in the shade but not in the sun. It seems that leaf tissue lost to herbivores in the shade would be too expensive to replace under the limiting light conditions of forest understory and thereby could lead to a negative carbon balance for the plant. It is in the deep shade scenario, under which *E. coccineum* seedlings apparently are close to their physiological tolerance limit, where herbivory may play an important role in plant establishment. This result adds to the small body of evidence on the possible contribution of herbivory to explain the distribution of a plant species along the light gradient [4], [5], [7], [53].

*E. coccineum* is one of the few tree species that is considered light-demanding in the southern temperate rainforest, where most woody species are somewhat shade-tolerant [54], [55]. Open sites in this temperate rainforest are becoming increasingly stressful during the plant growth season because of two drivers of global change. Thus, there is a marked decrease in summer precipitation (Saldaña *et al*., unpublished) and increased colonization by alien plant species (Godoy *et al*., unpublished).Results of the present study suggest that, in order to enhance shade-tolerance, *E. coccineum* must not only develop particular life history traits and physiological and morphological features (reviewed in [70]), but it should also evolve resistance or tolerance against herbivores.
